# Chitosan hydrogel-loaded MSC-derived extracellular vesicles promote skin rejuvenation by ameliorating the senescence of dermal fibroblasts

**DOI:** 10.1186/s13287-021-02262-4

**Published:** 2021-03-20

**Authors:** Xiangnan Zhao, Yue Liu, Pingping Jia, Hui Cheng, Chen Wang, Shang Chen, Haoyan Huang, Zhibo Han, Zhong-Chao Han, Krzysztof Marycz, Xiaoniao Chen, Zongjin Li

**Affiliations:** 1 Nankai University School of Medicine, 94 Weijin Road, Tianjin, 300071 China; 2 The Key Laboratory of Bioactive Materials, Ministry of Education, Nankai University, The College of Life Sciences, Tianjin, 300071 China; 3 Henan Key Laboratory of Medical Tissue Regeneration, Xinxiang Medical University, Xinxiang, 453003 China; 4 Jiangxi Engineering Research Center for Stem Cell, Shangrao, 334109 Jiangxi China; 5 Tianjin Key Laboratory of Engineering Technologies for Cell Pharmaceutical, National Engineering Research Center of Cell Products, AmCellGene Co., Ltd., Tianjin, 300457 China; 6 Beijing Engineering Laboratory of Perinatal Stem Cells, Beijing Institute of Health and Stem Cells, Health & Biotech Co., Beijing, 100176 China; 7 Department of Experimental Biology, Wroclaw University of Environmental and Life Sciences, Norwida 27B, 50-375 Wrocław, Poland; 8 Beijing Tongren Eye Center, Beijing Tongren Hospital, Capital Medical University, Beijing, 100730 China

**Keywords:** Extracellular vesicles (EVs), Chitosan hydrogel, Dermal fibroblasts (DFLs), Skin aging, Extracellular matrix (ECM)

## Abstract

**Background:**

The senescence of dermal fibroblasts (DFLs) leads to an imbalance in the synthesis and degradation of extracellular matrix (ECM) proteins, presenting so-called senescence-associated secretory phenotype (SASP), which ultimately leads to skin aging. Recently, mesenchymal stem cell (MSC)-derived extracellular vesicles (EVs) have been recognized as a promising cell-free therapy for degenerative diseases, which opens a new avenue for skin aging treatment.

**Methods:**

In this study, we utilized chitosan (CS) hydrogel for effective loading and sustained release of EVs. In vitro, we explored the rejuvenation effects of CS hydrogel-incorporated EVs (CS-EVs) on replicative senescence DFLs through a series of experiments such as senescence-associated β-galactosidase (SA-β-gal) staining, RT-PCR, and Western blot analysis. Besides, we employed local multi-site subcutaneous injection to treat skin aging of naturally aged mice with CS-EVs and DiI fluorescent dye was used to label EVs to achieve in vivo real-time tracking.

**Results:**

CS-EVs can significantly improve the biological functions of senescent fibroblasts, including promoting their proliferation, enhancing the synthesis of ECM proteins, and inhibiting the overexpression of matrix metalloproteinases (MMPs). Moreover, CS hydrogel could prolong the release of EVs and significantly increase the retention of EVs in vivo. After CS-EVs subcutaneous injection treatment, the aging skin tissues showed a rejuvenation state, manifested explicitly as the enhanced expression of collagen, the decreased expression of SASP-related factors, and the restoration of tissue structures.

**Conclusions:**

CS hydrogel-encapsulated EVs could delay the skin aging processes by ameliorating the function of aging DFLs. Our results also highlight the potential of CS hydrogel-encapsulated EVs as a novel therapeutic strategy for improving aging skin to rejuvenation.

## Introduction

Aging is an unavoidable process for everyone. The most evident and visible symptoms of aging in humans are first manifested by changing skin appearances, such as skin sagging, loss of elasticity, and wrinkle formation [1]. It is generally believed that skin aging is driven by various intrinsic and extrinsic factors, including various kinds of skin cell senescence [2]. Thereinto, dermal fibroblasts (DFLs) are the main skin stromal cells that secrete extracellular matrix (ECM). DFLs can synthesize and secrete ECM components such as collagen, elastin, and hyaluronic acid, which are processed to assemble fibers, giving the skin elasticity and toughness and helping to keep the juvenescence of skin [3]. However, during the aging processes, ECM is gradually degraded and disorganized, which will deleteriously alter the function of resident fibroblasts. In addition, several studies have provided convincing evidences that senescent fibroblasts markedly accumulate with age and thus might be detrimental to the skin [4, 5].

Cellular senescence is the basis of tissue and organism aging. Under the action of one or more triggering factors, cells break away from the cell cycle, present a “senescence-associated secretory phenotype” (SASP), secrete a variety of senescence information transmitting substances, and eventually irreversibly lose the ability to grow and proliferate [6]. In 2008, Coppe et al. proposed for the first time that senescent cells can promote the canceration of adjacent precancerous cells by secreting inflammation and oncogene-related factors, and defined this characteristic of senescent cells as SASP [7]. The accumulation of senescent cells in various organs is accompanied by a series of complex SASP. SASP includes pro-inflammatory cytokines (IL-1α, IL-1β, IL-6, and IL-8), growth factors (HGF, TGF-β), chemokines (CXCL-1/3 and CXCL-10), and matrix remodeling enzymes (MMPs-1, MMP-2, MMP-3) [8–11]. The senescence of skin tissue is often accompanied by the senescence of DFLs. Similarly, these senescent DFLs will also exhibit the above-mentioned SASP characteristics [4, 12, 13]. On the one hand, the aging DFLs have reduced ECM protein synthesis, such as collagen, elastin, and fibronectin [8]. On the other hand, the aging DFLs overexpress and secrete many pro-inflammatory and catabolic factors, such as pro-inflammatory cytokines and matrix metalloproteinases [10, 11]. More importantly, the continuous and excessive accumulation of senescent cells in tissues directly affects skin characteristics and accelerates the development of several age-related diseases, such as cancer [14–16]. Therefore, functional changes caused by fibroblast senescence are one of the key factors of skin aging [17].

Mesenchymal stem cells (MSCs) have been defined as multipotent stem cells with greater self-renewal and differentiation capabilities, which can be derived from several kinds of tissue types, such as the placenta, adipose, bone, and umbilical cord [6, 18, 19]. An increasing number of studies have revealed that MSCs possess significantly therapeutic potential for promoting wound healing [20–22], ameliorating myocardial infarction [23], and repairing ischemic diseases [24, 25]. Besides, MSCs have attracted great attention due to their large therapeutic potential in ameliorating skin aging [26, 27]. Studies have shown that adipose-derived stem cells (ADSCs) can promote skin regeneration through glycation inhibition and anti-oxidation in D-galactose-induced mouse skin aging models [26, 27]. Most importantly, the anti-aging application of MSCs for skin aging may be due to EV secretion through paracrine action in stem cells [28].

The successful delivery of drugs or active molecules to native tissues has become one of the promising approaches for optimal tissue repair and regeneration. Chitosan hydrogels (CS), with thermal sensitivity and loose porous structural properties, have been used as carriers for sustained release of drug [24, 29]. Furthermore, acting as an injectable hydrogel, CS can be administered by minimally invasive modality while incorporating EVs into target tissues for a better outcome [30].

In this study, we hypothesized that CS hydrogel-loaded EVs (CS-EVs) could exhibit beneficial effects on senescent fibroblasts to ameliorate skin aging. In order to simulate aging fibroblasts in vitro, we extracted fibroblasts from the skin tissue of suckling mice and established a replicative aging model of dermal fibroblasts through a continuous passage. Subsequently, we investigated the regulation of SASP in senescent fibroblasts by co-incubating with CS-EVs. Also, we evaluated the anti-aging effect of CS-EVs in natural aging mice through subcutaneous injection and explored the underlying mechanisms of tissue rejuvenation in skin aging.

## Materials and methods

### Cell culture

The human placental mesenchymal stem cells (hP-MSCs) were isolated as described previously [31] and were cultured in DMEM/F12 medium (Gibco, Grand Island, NY) with 10% bovine extracellular vesicle-free FBS (HyClone) and 100 U/ml penicillin–streptomycin (Gibco). EV-free FBS was obtained by ultracentrifugation (Beckman Coulter, Brea, CA) at 100,000*g* for 2 h at 4 °C [31–33]. The hP-MSCs used in subsequent experiments were all between passages 4 and 8.

### Dermal fibroblast (DFL) isolation

Mouse dermal fibroblasts were harvested as previously reported [34]. In brief, full-thickness skin harvested from newborn mice was treated with 1.2 U/mL Dispase II (Gibco; Invitrogen, Paisley, UK) for 12 h at 4 °C. Next, the epidermis is peeled off, leaving only the dermis layer. Then, use scissors to cut the dermis as much as possible. Then, the pieces were placed in phosphate-buffered saline (PBS) with 0.25% Trypsin (Sigma-Aldrich, St. Louis, MO, USA) and incubated at 37 °C for 10 min. After digestion, pieces were centrifuged at 1200 rpm for 10 min, resuspended in DMEM complete medium, and cultured at 37 °C in a humidified 5% CO_2_, 95% air incubator. After about 3 days, fibroblasts will crawl out of the edges of the dermal tissue.

### Replicative senescence model of DFLs

Cell replicative senescence is a commonly used experimental aging model used to illustrate the internal mechanism of organ aging [35]. In this study, we established a replicative senescence model of mouse primary DFLs by simulating the aging process of normal cells. In brief, the primary DFLs were isolated from the skin of newborn mice and cultured. The primary cells were passaged for 8 generations. A characteristic feature of aging DFLs is their slow growth rate.

### EV isolation

The method for extracellular vesicle isolation was performed as previously reported [31–33, 36]. Firstly, EV-free FBS was prepared by ultracentrifugation at 100,000*g* for 2 h at 4 °C to effectively deplete extracellular vesicles. The supernatant was subsequently filtered by a 0.22-μm filter (Millipore). Secondly, hP-MSCs were cultured in DMEM/F12 complete medium containing 10% FBS, 1% L-glutamine, 1% penicillin−streptomycin, 1% non-essential amino acids, and 87% DMEM/F12 basic medium. When the cell confluence reached about 80%, the medium was discarded and washed twice with PBS. The medium was replaced with DMEM/F12 medium containing 10% EV-free FBS, and the conditioned medium was collected after 24 h of continuous cultivation. Thirdly, the collected conditioned medium was centrifuged at 500*g* for 10 min to remove the cells in the medium; after centrifugation, the supernatant was collected and centrifuged at 2000*g* for 20 min to precipitate dead cells; then, the supernatant was collected. To remove cell debris, a 10,000*g* rotation speed was given for 30 min. Finally, continue to recycle the supernatant, centrifuge at 100,000*g* for 70 min, repeat this step, and collect the precipitate to be extracellular vesicles. EVs were used immediately or stored at − 80 °C.

### EV characterization

The typical morphology of the collected EVs was observed using transmission electron microscopy (TEM; Talos F200C, Hillsboro, OR). EVs were fixed in 1% glutaraldehyde solution for 5 min. EV samples were dehydrated with absolute ethanol for 10 min and were collected on formvar/carbon-coated copper grids (Zhongjingkeji Technology, Beijing, China). Then, the grids were incubated with 2% phosphotungstic acid for 5 min and washed with ddH2O. The grids were dried completely and imaged using TEM. A BI-200SM laser scattering instrument (ZetaPALS, Brookhaven, NY) was used to analyze the EV concentration and particle size by dynamic light scattering (DLS) measurements at 20 °C. The Brownian motion of each particle was tracked and analyzed, and the hydrodynamic diameter and concentration of nanoparticles were calculated by combining the Stokes–Einstein equation. Analyze the marker proteins on the surface of EVs employing western blots, such as CD9 (1:1000, Abcam, Cambridge, UK), CD63 (1:1000, Abcam, USA), and TSG101 (11,000, Abcam).

### CS hydrogel properties

According to the previous literature, we prepared CS hydrogel [29, 30]. Thermo-responsive chitosan hydrogel was tested under different temperature conditions. The chitosan powder was dissolved in 0.1 M acetic acid, sterilized through a 0.22-μm filter, prepared into a 2% chitosan stock solution, and stored at 4 °C. The 50% β-glycerophosphate (β-GP) solution was added to the CS solution at a volume ratio of 5:1 and stirred continuously in an ice bath until the two solutions were completely mixed. After incubating at 37 °C for 30 min, the CS solution could cross-link into the hydrogel. CS hydrogel was freeze-dried under vacuum for 2 days, and the surface morphology and void size were observed under a scanning electron microscope (SEM; HITACHI X-650, Tokyo, Japan) after gold spraying.

### Preparation of CS hydrogel-encapsulated EVs

In order to obtain CS hydrogel-incorporated EV (CS-EVs), 75 μg EVs were mixed with equal volume 2% CS solution; after adding β-GP, the above-mixed solution was incubated at 37 °C for 30 min. The CS-EVs solution could cross-link into the hydrogel [30].

### Release kinetics of CS-EVs

In order to measure the release rate of EVs in vitro, EVs were labeled with Gluc-lactadherin, a fusion protein of Gaussia luciferase (Gluc reporter protein) and lactadherin (an EV-tropic protein) as previously reported [33]. In brief, hP-MSCs were transfected with lentiviruses of Gluc-lactadherin and hP-MSC-derived EVs were assessed for concentration-dependent expression of Gluc bioluminescent signals. IVIS Lumina imaging system was used to measure the release rate of EVs [30]. In brief, 100 μg of Gluc-labeled EVs was resuspended in 200 μL of chitosan/β-glycerophosphate solution and incubated in a 48-well plate at 37 °C for 30 min to form the hydrogel. Then, add 200 μL of PBS to each well to submerge the hydrogel encapsulating with EVs. After incubating in a 37 °C hood, the supernatant PBS was collected and transferred to another 48-well plate for BLI analysis at different time points.

### EV internalization

According to previous reports, EVs are labeled with CM-DiI membrane dye (Invitrogen, Carlsbad, CA) [31]. Briefly, 50 μg EVs were mixed with 10 μL CM-DiI diluted in PBS (50 mL) and incubated at room temperature for 5 min. Unbound dye was removed by ultracentrifugation at 100,000*g* for 2 h at 4 °C; then, the pellets were resuspended in PBS and washed three times. DiI-labeled EVs were co-cultured with DFLs for 24 h. The second day, DFLs were washed with PBS and fixed in 4% PFA solution. The nuclei were stained with DAPI, and the uptake was observed under fluorescence microscopy (Nikon).

### MTT assay

The beneficial effects of EVs on cell proliferation and anti-apoptosis were assessed by MTT assay (Sigma) following the manufacturer’s protocol. Briefly, DFLs (1 × 10^3^ cells/well) were cultured in a 96-well plate for 24 h. On the next day, adding different concentrations of H_2_O_2_ or (and) EVs to the medium, with 5 repeating wells in each group. After 24 h and 48 h, 5 × MTT was diluted to 1 × MTT with dilution buffer. Add 150 μL DMSO to each well and shake with a shaker, followed with incubation with 50 μL 1 × MTT for 4 h at 37 °C. The optical density of each well can be measured at a wavelength of 490 nm using a microplate reader (Promega).

### Scratch wound healing assay

The effect of CS-EVs on the migration of DFLs was determined by scratch wound assay as described in the previous study [37]. 1 × 10^5^ DFLs were seeded into a 6-well plate containing DMEM/F12 complete medium. When DFLs reached 70–80% confluence, scratch wounds were generated across each well using a sterile plastic 10-μL micropipette tip. After washing the cells with PBS, EVs with different concentration gradients were added. Images were taken at 0 and 12 h by an inverted microscope (Olympus, Lake Success, NY). Scratched areas were measured using the Image-Pro Plus 6.0 software.

### Senescence-associated β-galactosidase (SA-β-gal) assay

Cells and frozen sections were stained with SA-β-Gal Staining Kit (Beyotime Biotechnology, China) following the manufacturer’s protocol. Briefly, cultured cells and slides were fixed and then incubated at 37 °C with a staining solution containing 1 mg/mL of 5-bromo-3-chloro-4-indolyl β-D-galactoside (X-gal) overnight.

### Ki67 immunofluorescence

Senescent fibroblasts were incubated with an FBS-free medium in the presence of EVs or CS-EVs for 24 h. PBS and CS served as the control. After washing by PBS, the cells were fixed with 4% formaldehyde (Sigma-Aldrich) and then blocked with 10% BSA for 2 h. Then, the cells were incubated with the primary antibody against Ki67 (Abcam, Cambridge, MA, USA) at 4 °C overnight, followed by appropriate fluorescently labeled secondary antibodies (Life Technologies, Carlsbad, CA). Nuclei were stained with DAPI (Sigma) for 5 min. The number of Ki67-positive cells in three random fields in each group was measured by ImageJ software.

### Quantitative real-time PCR

RNA extraction from cells and skin tissues was performed using Trizol reagent (Invitrogen, Grand Island, NY) referring to the manufacturer’s manuals. Afterwards, the obtained RNA was converted to cDNA using the BioScript All-in-One cDNA Synthesis SuperMix (Bimake, Houston, TX). Real-time RT-PCR was performed with FastStart Universal SYBR Green Master (Roche, Mannheim, Germany) by Opticon® System (Bio-Rad, Hercules, CA). Data were analyzed by the 2^−ΔΔCt^ method. Primer sequences are listed in Suppl. Table 1.

### Establishment and treatment of the natural aging mouse model

Female FVB mice (48 weeks old) were used in this study. Mice were anesthetized with avertin (2.5%, 240 mg/kg), then shaved the hair of the dorsal surface with an electric clipper. Three points were selected on the dorsal skin for local multi-site subcutaneous injection. Specifically, 75 μg of EVs suspended in PBS or CS hydrogel was injected into the dorsal skin at a 100-μL total volume. Equivoluminal injections of PBS or chitosan hydrogel served as the control. Furthermore, the method of cumulative three injections was adopted at days 1, 7, and 14. Animal experiments were conducted after approval by the Animal Care and Use Committee in Nankai University.

### Tracking of EVs

In order to monitor the retention of EVs locally delivered in vivo in real-time, IVIS Lumina imaging system (Xenogen Corporation, Hopkinto, MA) was used to image DiI-labeled EVs or CS-EVs 24 h after subcutaneous injection. The light at 535 nm and 565 nm is used as the excitation wavelength and emission wavelength, respectively. At the indicated time points, we imaged the retention rate of EVs by bioluminescence imaging (BLI). The intensity of fluorescence signals was quantified by average radiance from a fixed-area region of interest (ROI) over the skin area.

### Histological analysis

On 21 days after the first treatment, all mice were euthanized and skin samples were harvested. The excised skin tissue samples were immediately fixed with 4% paraformaldehyde (PFA) and then embedded in paraffin. Hematoxylin–eosin (H&E) staining was performed to observe the structural changes of skin tissue. In order to evaluate the expression of collagen fibers in aging skin tissues after CS-EVs therapy, Masson’s staining was performed. Immunohistochemistry staining was conducted to detect the expression of vimentin (Santa Cruz Biotechnology). The images were analyzed by ImageJ software.

### Statistical analysis

The statistical analysis and graphs were generated using GraphPad Prism 5.0 statistical software (GraphPad software, Inc., San Diego, CA) via one- or two-way ANOVA for multiple comparisons and *t* test for two group comparisons. All results are expressed as mean ± SD. *P* < 0.05 was considered statistically significant.

## Results

### Characterization of EVs and CS hydrogel

**Fig. 1:**
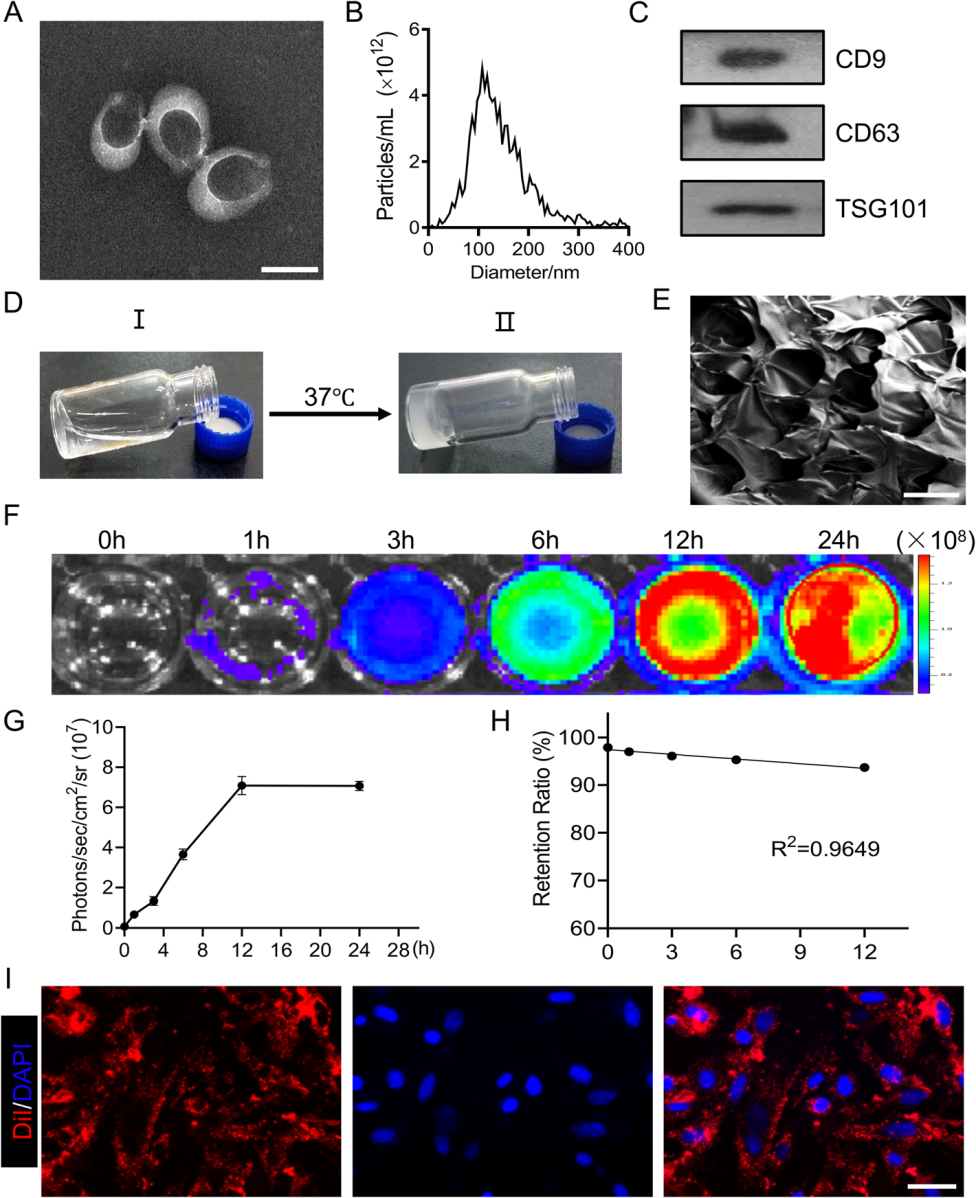
Characterization of MSC-EVs and thermosensitive chitosan hydrogel. **a** TEM photomicrograph of EVs. Scale bar, 100 nm. **b** Size distribution of MSC-EVs was measured by NTA in scatter mode. **c** The protein markers of MSC-EVs were detected by Western blotting. **d** Optical images of the chitosan solution (I) and hydrogel (II). **e** SEM photograph of chitosan hydrogel. Scale bar, 100 μm. **f**, **g** The released EVs were measured by quantitative analysis of Gluc signal. **h** Retention ratio of EVs in CS-EVs after immersing in PBS within 12 h (*R*^2^ = 0.9649). The signal activity was represented by photons/s/cm^2^/steradian. **i** Dermal fibroblast cells could uptake CM-DiI-labeled EVs (red). Scale bar, 100 μm

We isolated EVs from the conditioned medium of hP-MSCs by ultracentrifugation. The characterization of EVs was demonstrated by TEM, DLS, and Western blot analysis. The TEM image revealed that the morphology of EVs was cup-shaped round bilayer membrane vesicles with a diameter of about 100–120 nm (Fig. 1a). The size of EVs was determined by DLS, and the results showed that the average particle diameter is about 120 nm (Fig. 1b). As shown in the western blotting analysis, we confirmed the expression of CD9, CD63, and TSG101 in EVs, which are the surface markers of EVs (Fig. 1c). These data indicated that we have successfully isolated MSC-derived EVs.

In order to further improve the stability and persistence of EVs, we used CS hydrogel as an ideal injectable carrier to achieve sustained-release EVs. Firstly, the chitosan neutralized with β-GP was liquid at 4 °C and cross-linked into the hydrogel when the temperature rose to around 37 °C (Fig. 1d). Moreover, scanning electron microscopy (SEM) showed that the freeze-dried chitosan hydrogel had a loose porous structure with the average mesh size of about 80 μm (Fig. 1e), which contributes to its sustained release effect.

We incorporated EVs into this injectable CS hydrogel to form CS-encapsulated EVs (CS-EVs), which could continuously release EV to the surrounding environment. The concentration of EVs in the supernatant was determined by the BLI method to further detect the release profile of EVs (Fig. 1f, g). According to the trend of linear correlation between the number of EVs and Gluc signal (Figure S1), we can calculate the concentration of EVs in the PBS supernatant. Calculations show that within 12 h, 100 μg of EVs encapsulated by CS hydrogel can continuously release 0.25 μg of EVs per hour into the surroundings. Therefore, the retention rate of EVs in the CS hydrogel can be determined (Fig. 1h). These data indicate that CS hydrogel can effectively embed EVs and achieve sustained release EVs in vitro.

### Isolation of primary mouse dermal fibroblasts and internalization of EVs

It is well known that DFLs can synthesize and secrete ECM components such as collagen, elastin, and hyaluronic acid, which are of great significance for maintaining the elasticity and toughness of the skin. The aging of skin tissue is often accompanied by the aging of DFLs. In order to further understand the functions and characteristics of DFLs, we isolated DFLs from the skin of suckling mice. We cut the whole layer of the newborn mouse skin and then separated the epidermis and dermis in a dish. After digestion with neutral protease and adherent culture for 3 days, we observed that fibroblasts migrated from the edge of the dermal tissue (Figure S2).

In addition, to further determine that EVs can be efficiently uptaken by DFLs in vitro, EVs were labeled with CM-DiI dye (red) and co-incubated with fibroblasts. After 24 h, we found that the labeled EVs co-localized with fibroblasts, mainly located in the perinuclear region (Fig. 1i). Overall, the results indicated that DiI dye was highly specific, reliable for labeling EVs and DiI-labeled EVs were successfully internalized by DFLs in vitro.

### Dermal fibroblast replicative senescence and function changes

**Fig. 2:**
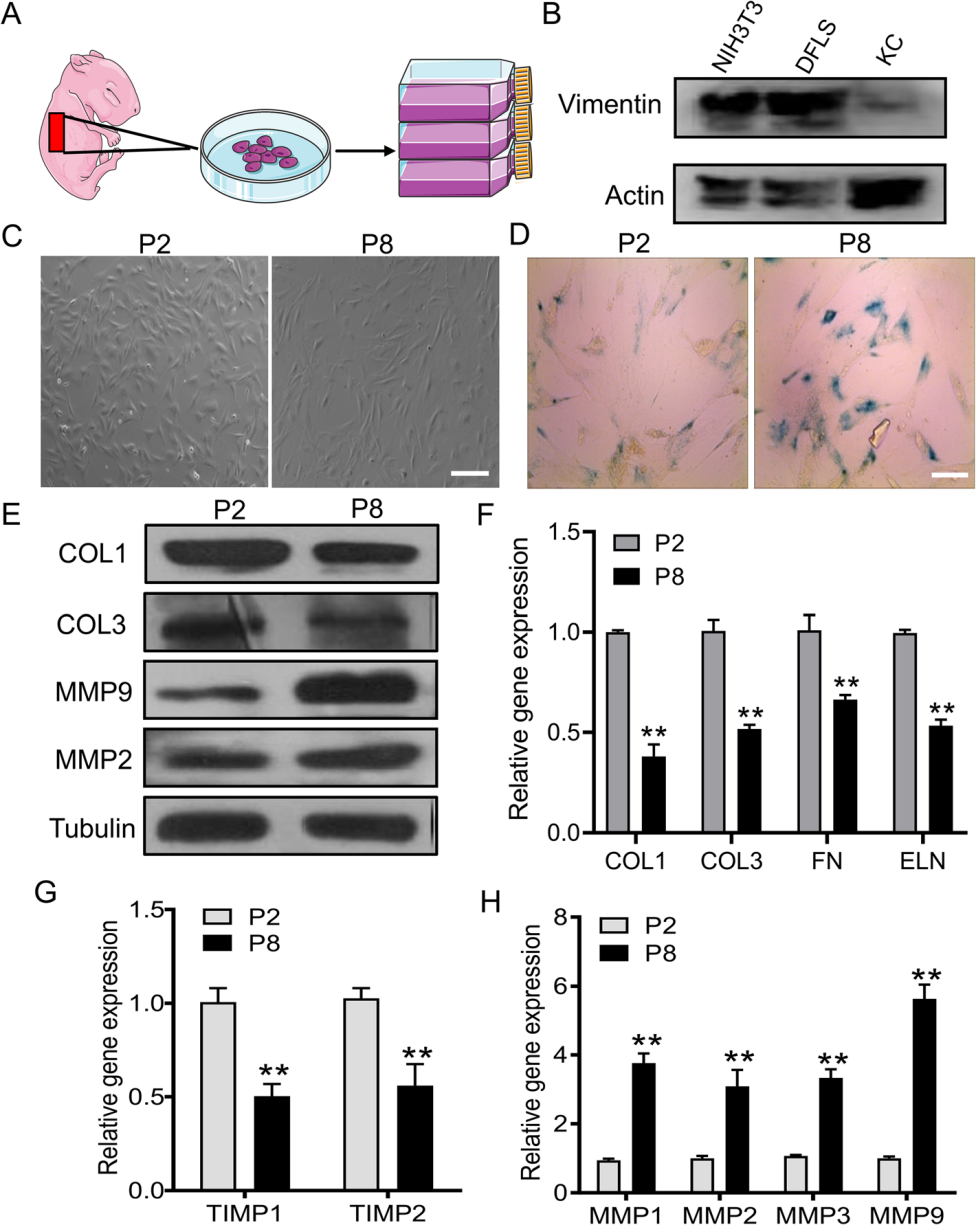
Construction and characterization of the replicative senescence model of dermal fibroblasts. **a** Schematic diagram of skin dermal fibroblast isolation. **b** The extracted dermal fibroblasts (DFLs) were verified by Western blotting, using mouse embryonic fibroblasts (NIH3T3) as a positive control and mouse skin keratinocytes (KC) as a negative control. **c** The morphological comparison of fibroblasts in early generation (P2) and replicative senescence (P8). Scale bar, 200 μm. **d** Detection of β-galactosidase activity in replicative senescence fibroblasts. Scale bar, 50 μm. **e** Western blot analysis of the expression of ECM proteins (COL1 and COL3) and matrix metalloproteinases (MMP9 and MMP2). **f**–**h** Real-time qPCR analysis of aging-related gene expression. Data are presented as the mean ± SD (*n* = 3; *P < 0.05, **P < 0.01 vs P2)

Most molecular hallmarks and function analysis of cellular senescence need to be identified in studies of skin aging in vitro by driving them into replicative. In this study, we therefore isolated primary mouse DFLs from newborn mouse skin tissue via tissue-block cultivation (Fig. 2a). Keratinocytes (KC) and DFLs are the main components of the skin, and keratinocytes can easily be mixed in the extraction process of DFLs. To verify that the isolated cells were DFLs, Western blot analysis of vimentin was performed. Vimentin is a marker of fibroblasts, and KC cells do not express. NIH3T3 was used as the positive control and keratinocytes as the negative control. The results showed that DFLs and NIH3T3 cells express a high level of vimentin, while KC not, which indicated the successful isolation of DFLs (Fig. 2b). Then, during the process of continuous passage, we ensured that cells were studied at late passages (≥ 8). The P8 DFLs varied in size and shape, the cytoplasm began to be granular, and debris was formed in the medium, which indicate the senescence (Fig. 2c). We observed higher SA-β-Gal expression, the marker of cellular senescence, in P8 DFLs (Fig. 2d). Therefore, P8 dermal fibroblasts can be used as a cellular senescence model for further in vitro experiments.

To investigate the biological function changes of senescent fibroblasts, we examined different cell characteristics associated with the expression of ECM, matrix metalloproteinases (MMPs), and tissue inhibitor of metalloproteinases (TIMPs). Compared with P2 DFLs, the protein levels of collagen 1 (COL1) and collagen 3 (COL3) were significantly reduced in P8 DFLs, while the protein expression of MMP2 and MMP9 were significantly increased (Fig. 2e, Figure S3). Similarly, the transcript levels of COL1, COL3, fibronectin (FN), and elastin (ELN) were decreased in senescent P8 DFLs, as well as TIMP1 and TIMP2 (Fig. 2f, g). Conversely, the expression levels of matrix-degrading enzymes, MMP9 and MMP2, were clearly increased in senescent fibroblasts. Relative expression of MMP1, MMP2, MMP3, and MMP9 mRNA also obviously increased in P8 DFLs compared with the young generation (Fig. 2h). Taken together, these data indicated that senescent P8 dermal fibroblasts exhibited abnormal cell function.

### The anti-senescent effect of CS-EVs on senescent fibroblasts in vitro

**Fig. 3:**
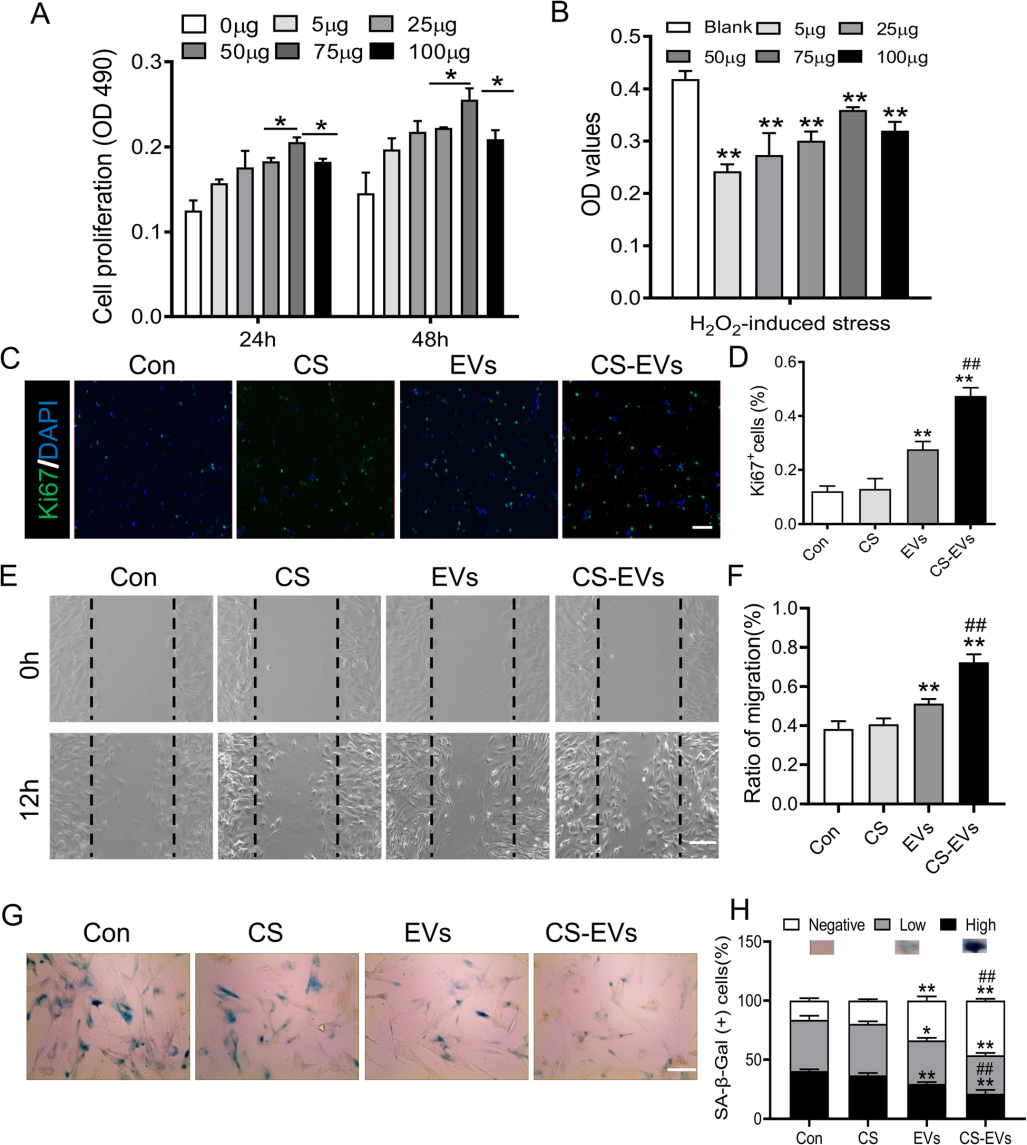
CS-EVs exert an anti-senescence effect on senescent fibroblasts and improve their biological function. **a** The optimal EV concentration for the highest viability of fibroblasts is 75 μg/mL. **b** Survival of fibroblasts in the presence of different concentrations of EVs under challenge from H_2_O_2_-induced stress (*n* = 3; *P < 0.05 vs blank). **c** Representative images showed the proliferation (Ki67, green) of fibroblasts incubated with 75 μg/mL EVs or CS-EVs for 24 h. Scale bar, 200 μm. **d** The statistics of Ki67-positive cells in each group. **e** Scratch wound healing assay of DFLs treated with 100 μg/mL EVs or CS-EVs. Scale bar, 200 μm. **f** Migration ratio statistics of fibroblasts in each group. **g** Effects of EVs or CS-EVs on SA-β-gal activity of fibroblasts. Scale bar, 50 μm. **h** The percentage of SA-β-gal-positive cells. Data are presented as the mean ± SD (*n* = 3; *P < 0.05, **P < 0.01 vs Con; ^#^*P* < 0.05 vs EVs)

To further investigate whether CS-EVs could ameliorate multiple phenotypes associated with cellular senescence, here, we first detected the cell-protective capacity of EVs. MTT assay revealed that EVs promoted senescent fibroblast proliferation with an increase of EV concentration at 24 and 48 h and the peak was 75 μg/mL (Fig. 3a). In order to test the anti-apoptosis effects of EVs, fibroblasts were treated with 500 μM hydrogen peroxide (H_2_O_2_), and cell survival was monitored by MTT. Results show that EVs ameliorated senescent fibroblast survival in a dose-dependent manner (Fig. 3b).

Cell proliferation of fibroblasts was investigated by proliferating cell nuclear antigen (Ki67) staining. Fibroblasts were incubated with 75 μg/mL EVs or CS-EVs, and the control group was treated with PBS or CS at the same volume. Our results indicated that the percent of Ki-67^+^ cells were markedly higher in the presence of CS-EVs (Fig. 3c, d). Besides, scratch assay revealed that CS-EVs could increase the migration of DFLs (Fig. 3e, f). All these data suggested that CS-EVs can effectively promote the proliferation and migration of senescent fibroblasts in vitro. Furthermore, we examined whether CS-EVs could reverse cellular senescence. CS-EVs treatment obviously reduced the percentage of SA-β-Gal-positive cells (21%) compared with the control group (40%) (Fig. 3g, h).

### CS-EVs promoted the synthesis of ECM

**Fig. 4:**
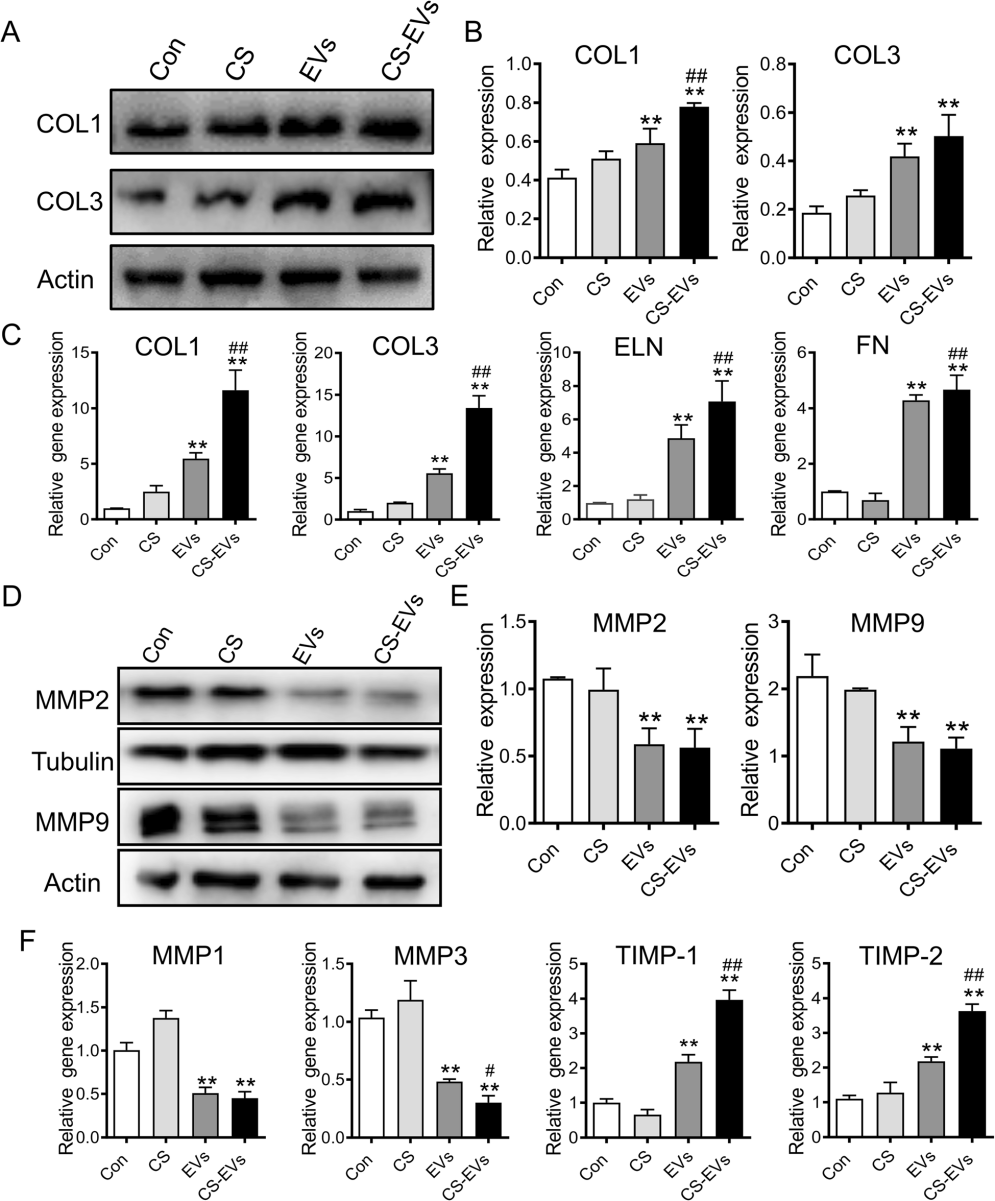
CS-EVs promoted the accumulation of ECM in senescent fibroblasts. **a** Protein levels of Col1 and Col3 were detected by Western blotting in fibroblasts incubated with 75 μg/mL EVs or CS-EVs for 24 h. **b** Quantification of protein levels normalized to β-actin. **c** RT-PCR analysis of the expression levels of ECM-associated genes in fibroblasts treated with EVs or CS-EVs for 24 h. **d** Protein levels of MMPs were detected by Western blotting in fibroblasts incubated with EVs or CS-EVs for 24 h. **e** Quantification of protein levels normalized to Tubulin and β-actin, respectively. **f** RT-PCR analysis of the expression levels of MMP and TIMP genes in fibroblasts treated with EVs or CS-EVs for 24 h. Data are presented as the mean ± SD (*n* = 3; *P < 0.05, **P < 0.01 vs Con; ^#^*P* < 0.05 vs EVs)

To investigate the effect of CS-EVs associated with ECM synthesis, Western blot analysis revealed the upregulation of COL1 and COL3 (Fig. 4a, b). CS-EVs also could elevate the expression of ECM molecules including COL1, COL3, ELN, and FN by RT-PCR analysis (Fig. 4c). Moreover, pretreatment with CS-EVs could significantly inhibit the expression of matrix-degrading enzymes (MMP-1/3/2/9) (Fig. 4d–f). In addition, CS-EVs could be able to restore the expression of tissue inhibitor of metalloproteinases (TIMP-1/2) (Fig. 4f). Collectively, CS-EVs could increase ECM synthesis and tissue regeneration through promoting the expression of related proteins and TIMP as well as inhibiting MMPs in naturally senescent fibroblasts.

### CS hydrogel enhanced the retention of EVs in vivo

**Fig. 5:**
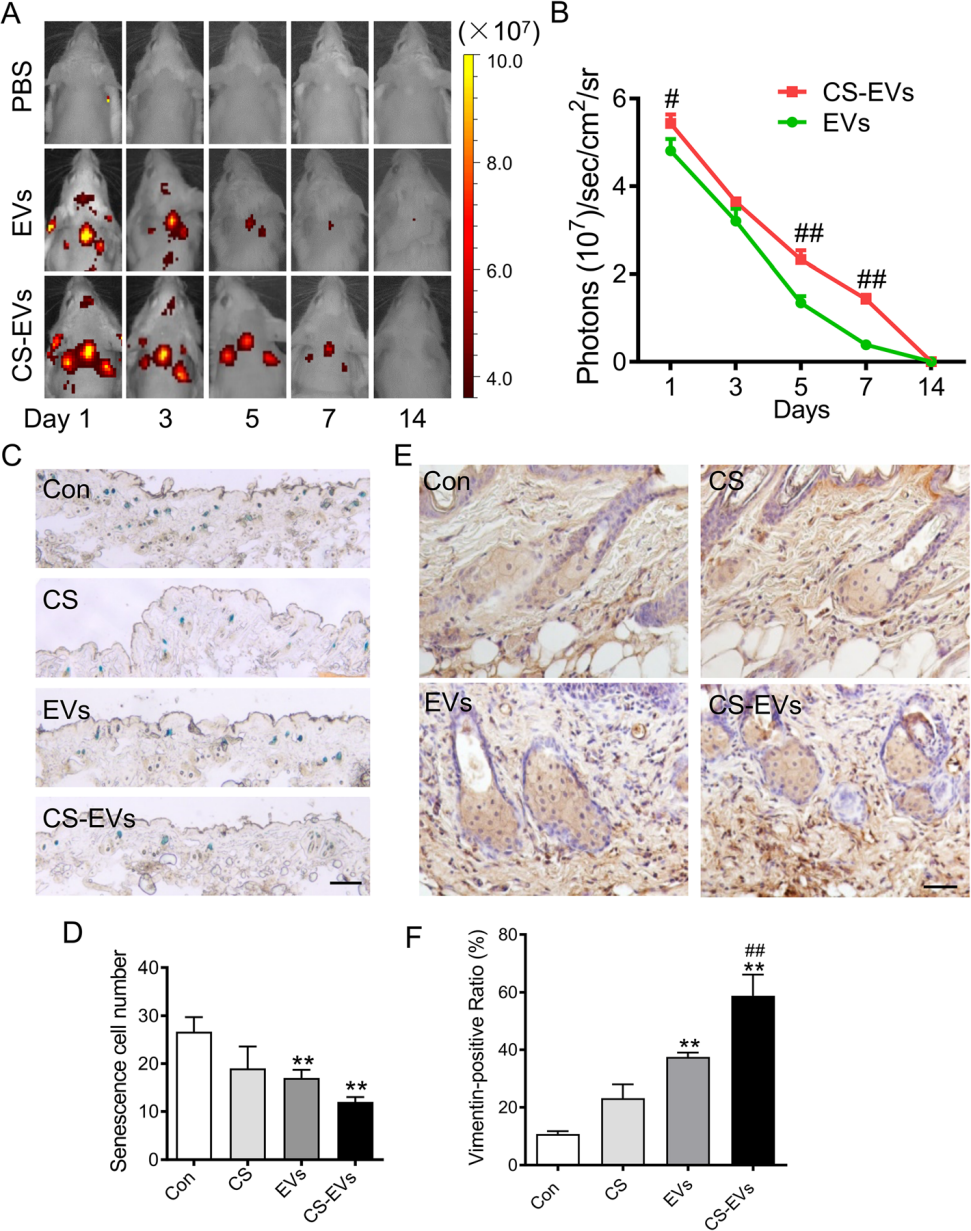
Improved senescent state of fibroblasts in aging skin by CS-EVs. **a** Representative time-dependent in vivo fluorescence images of DiI-labeled EVs and CS-EVs following local injection in mouse dorsal skin. **b** Quantitative analysis of DiI signals of EVs in senescent murine dorsal skin at different points. The signal activity was represented by photons/s/cm^2^/steradian (*n* = 3; ^#^*P* < 0.05, ^##^*P* < 0.01 vs EVs). **c** Detection of fibroblast β-galactosidase activity in natural aging mouse skin. Scale bar, 50 μm. **d** The quantification of SA-β-gal-positive cells. **e** Representative images of vimentin (fibroblast biomarker) staining showed the cell proliferation treated with EVs or CS-EVs on day 21. Scale bar, 150 μm. **f** The quantification of vimentin-positive cell percentage. Data are presented as the mean ± SD (*n* = 3; *P < 0.05, **P < 0.01, ***P < 0.001 vs Con; ^#^*P* < 0.05, ^##^*P* < 0.01 vs EVs)

To track the retention of EVs in vivo, 75 μg of DiI-labeled EVs incorporated with CS hydrogel (CS-EVs) or suspended in PBS was subcutaneous injected into the dorsal skin of the natural aging mouse at a 100-μL total volume. Subsequently, DiI-labeled EVs or CS-EVs were imaged at the indicated time points after treatment using an IVIS Lumina imaging system. The bioluminescence imaging (BLI) data exhibited a stronger signal from CS hydrogel application (Fig. 5a, b), suggesting that the incorporation with CS hydrogel could augment EV retention and might provide a way to increase the therapeutic potential of EVs.

### CS-EVs reversed the senescence state of fibroblasts in aging skin

To explore the effect of CS-EVs on fibroblasts in aging skin, natural aging mice were classified into four groups, PBS, CS, EVs, and CS-EVs. Seventy-five micrograms of EVs suspended in PBS or chitosan hydrogel (2% CS mixed with 75 μg EVs equal volume) was injected into the dorsal skin at a 100-μL total volume. Equivoluminal injections of PBS or CS hydrogel served as the control. Three time points at days 1, 7, and 14 were selected on the dorsal skin for local multi-site subcutaneous injection (Figure S4). Senescence-associated β-galactosidase (SA-β-gal) revealed a significant reduction in the number of senescent fibroblasts within the skin stroma with treatment of CS-EVs compared with controls (Fig. 5c, d). In addition, the immunohistochemical staining revealed the upregulation of vimentin with the treatment of CS-EVs (Fig. 5e, f). Together, these data indicated that CS-EVs could be able to reverse the senescence state of fibroblasts in aging skin.

### CS-EVs stimulated collagen remodeling and skin regeneration in the aging mouse

**Fig. 6:**
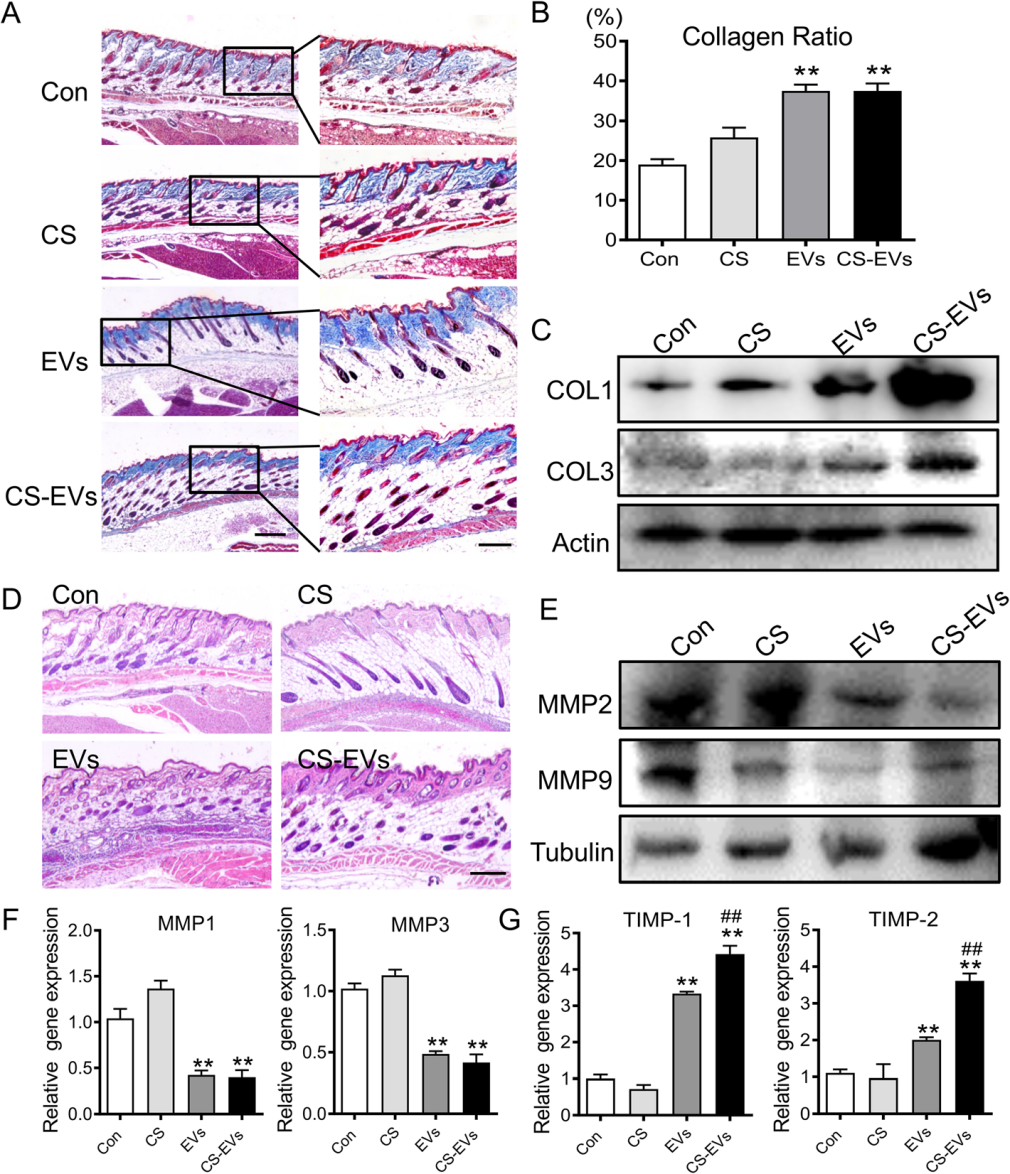
Treatment of CS-EVs accelerates skin remodeling. **a** Histologic images of collagen remodeling by Masson trichrome staining. Scale bar, 150 μm. Boxed areas are shown at higher magnification. **b** Quantitative statistics of collagen fibers in each group. **c** Western blot analysis of COL1 and COL3 protein expression. **d** Histologic images of skin appendage regeneration by HE staining. Scale bar, 100 μm. **e** Protein expression of MMPs were detected by Western blotting in therapeutic skin tissue. **f** Gene expression level of MMPs in aging skin. **g** Gene expression level of TIMPs in aging skin with EVs or CS-EVs treatment was detected by RT-PCR. Data are presented as the mean ± SD. *P < 0.05, **P < 0.01 vs Con; ^#^*P* < 0.05 vs EVs. All experiments were performed in triplicate

One of the well-known features of skin aging is collagen degradation. In aging mouse skin, collagen bundles decreased and became loose (Figure S5A, B). We prospected that beneficial effects of the CS-EVs may be directly involved in promoting collagen deposition and remodeling. Therefore, we investigated whether subcutaneous injection of CS-EVs could affect collagen production in the aging mouse. Masson’s trichrome staining showed increased collagen bundles in the CS-EV-treated group (Fig. 6a, b). More specifically, the morphology of the collagen bundles in control and CS groups were loose, broken, and disordered while the CS-EVs and EVs groups were dense, thickened, and highly ordered by comparison. Meanwhile, Col1 and Col3 expressions in the skin were upregulated significantly in the CS-EVs-treated group (Fig. 6c). As for skin structure, dorsal skin in an old mouse showed atrophy of the epidermis and dermal thickening (Figure S5A, B). Although HE staining results showed no significant changes in epidermis and dermis thickness after treatment, CS-EVs intervention could promote the regeneration and reconstruction of skin appendages such as hair follicles as well as sebaceous glands to rejuvenate the aging skin (Fig. 6d). Additionally, we further evaluated the altered expression of matrix metalloproteinases after treatment in aging skin. The expression of matrix-degrading enzymes (MMP-2,9,1,3) was decreased in the CS-EVs treatment group, contrary to the results with the other groups (Fig. 6e, f). However, the tissue inhibitor of metalloproteinases (TIMP-1,2) level was significantly elevated for the utilization of CS-EVs (Fig. 6g). These findings suggested that CS-EVs restored the altered expression of matrix-degrading enzymes in aging skin tissue. Taken together, CS-EVs exerted an anti-aging effect by promoting the remodeling of ECM*.*

## Discussion

**Fig. 7:**
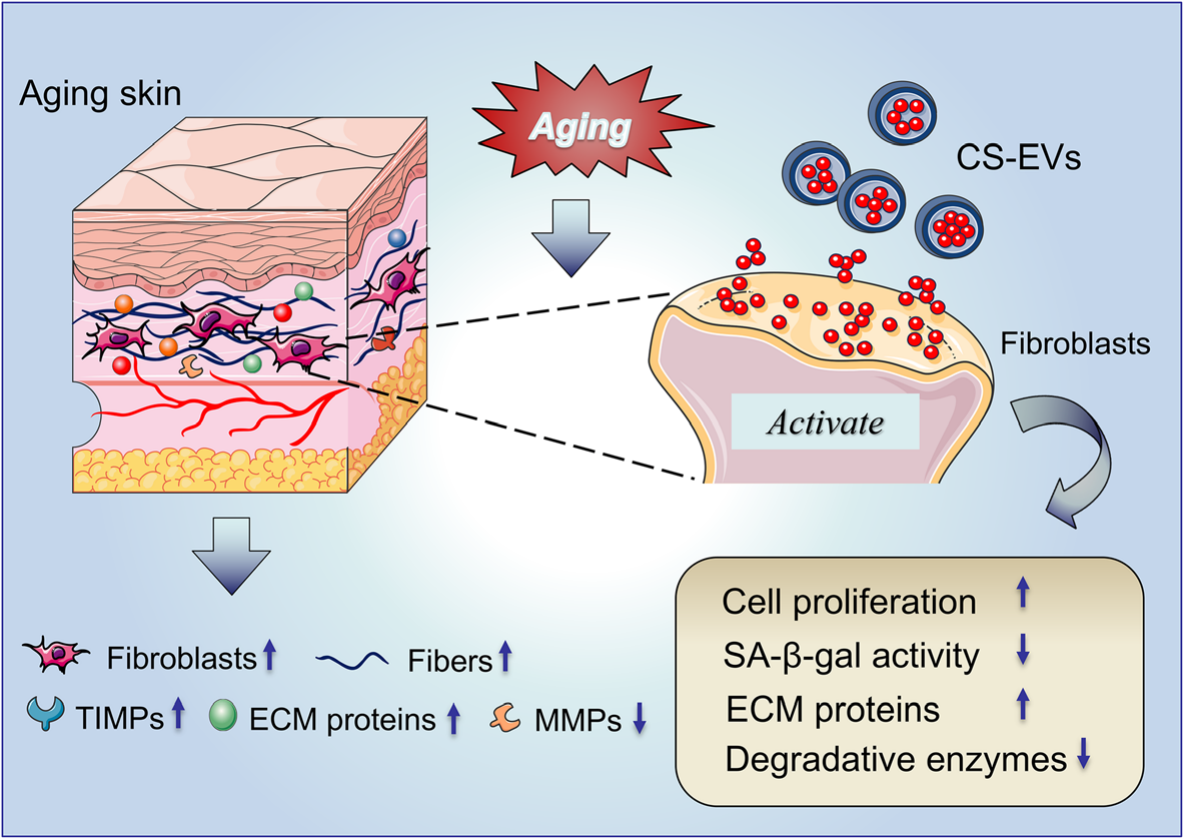
Schematic illustration of the role of CS-EVs on skin rejuvenation. The CS hydrogel load with EVs, a biological particle released by hP-MSC, to the target cells and activate senescent dermal fibroblasts. EVs incorporated with CS hydrogel could activate senescent fibroblasts by improving cellular proliferation, decreasing SA-β-gal activity, promoting ECM synthesis, and inhibiting ECM degradation. Consequently, CS-EVs therapy leads to improved functional recovery to ameliorate skin aging

We here demonstrated that CS-EVs could rejuvenate senescent dermal fibroblasts, thereby alleviating skin aging. Firstly, we successfully extracted primary DFLs from the skin of suckling mice, established a replicative senescence model in vitro by continuous passage, and revealed the dysfunctional characteristics of DFLs after aging. Secondly, we encapsulated MSC-EVs into CS hydrogel and applied them to senescent DFLs in vitro. Our results indicated that CS-EVs could rejuvenate senescent DFLs, as well as increase the proliferation and migration of DFLs, decrease the SA-β-Gal activity in DFLs, and enhance ECM protein synthesis of DFLs. Thirdly, our results revealed that CS hydrogel could significantly enhance the retention and stability of EVs as confirmed by BLI. In summary, CS-EVs displayed better anti-senescence effects on the functional and structural restoration of skin aging (Fig. 7).

The skin is the organ with the largest area of the human body exposed to the external environment, and skin aging is one of the main manifestations of human aging [38, 39]. Skin senescence often leads to the occurrence and development of various skin diseases. For example, the incidence of skin cancer will increase gradually with age [40, 41]. Dermal fibroblasts are one of the important cells in skin tissue. Decreased cell activity of DFLs is an important manifestation of skin aging processes and has been widely used in experimental research on skin aging [42–44]. The function of senescent fibroblasts is impaired, and the ability to synthesize collagen is reduced. At the same time, the high expression of MMP can specifically degrade almost all ECM components and destroy the normal structure of collagen fibers and elastic fibers [45–48]. Other ECM proteins, including elastic and fibronectin, also change generated from senescent fibroblasts, ultimately leading to a reduction in the amounts of functional cells [49].

Stem cells are seed cells for the renewal of various tissue cells and can secrete a variety of biologically active factors through paracrine function to promote damage repair [50]. EVs are one of the most important paracrine factors of stem cells. They carry many biologically active components in stem cells, including DNA, RNA, and protein, as a cell-free therapeutic agent in the field of anti-aging showing unique application prospects [51–53]. Studies have shown that embryonic stem cell (ESC)-conditioned medium and ESC-EVs can restore the vitality of senescent hP-MSCs [54]. A recently published study showed that EVs derived from umbilical cord MSCs rejuvenate aging adult bone marrow MSCs by transferring PCNA [55]. Together, these studies suggest that EVs from the stem cell could be good candidates for therapeutic strategies against aging.

Although we know the beneficial effects of stem cell-derived EVs on skin aging, low retention and stability remain an obstacle for clinical applications [30, 33]. More importantly, it is necessary to develop a valid method for EV-based therapeutics. As a carrier system with high biocompatibility, biomaterials can improve the survival rate of transplanted cells and can also imitate the ECM microenvironment to provide an ideal niche for implanted cells and drugs [56–58]. CS have been deeply studied and widely applicated in the field of biomedicine as a natural polymer material with excellent properties [29, 58]. Previous studies have confirmed that combining EVs from hP-MSC with injectable CS hydrogel can significantly retain EVs in the ischemic site of hind limbs. More interestingly, the CS hydrogel maintains the stability of EV protein and miRNA under physiological conditions, thereby greatly promoting the therapeutic effect of EVs [30]. Therefore, we chose CS hydrogel as the carrier of sustained-release EVs in this study.

At present, the research models of skin DFL senescence are mainly photoaging models, and there are still few studies on the replicative senescence of DFLs with MSC-EVs. In this study, a cell replicative senescence model was established through continuous passage, that is, a natural aging model, to study the improvement effect of CS-EVs on naturally aging skin fibroblasts, and further applied to naturally aging mice to verify its therapeutic effect. There are also some limitations to this study. We revealed that CS-EVs could rejuvenate aging DFLs and slow down skin aging-related properties in the aged mice. However, the specific mechanism by which CS-EVs can restore the vitality of senescent DFLs has not been further explored. Besides, the design and use of smart hydrogels for regenerative medicine is still an important field with huge potential and still needs to be fully investigated.

In conclusion, we prepared CS hydrogel-loaded EVs from hP-MSCs and examined their effects in the naturally aging mouse model. Our results revealed that CS-EVs could increase the proliferation, migration, and anti-senescence-related gene expressions in naturally senescent fibroblasts. Mounting evidences support that dermal fibroblasts mediate many changes in the ECM synthesis during skin aging to facilitate the occurrence of senescence phenotype. Our data indicate that ECM regeneration in senescent fibroblasts could be promoted, which may be at least partially due to the decrease in MMPS levels and accompanied by a corresponding increase in the levels of TIMPs under the treatment of CS hydrogel-loaded EVs. In brief, fibroblasts in aging skin recovered their function in terms of fibroblast-regulated ECM production largely when in response to the EVs. These findings provide a novel mechanism for how EVs play a role in skin aging.

## Supplementary Information

**Additional file 1: Table S1.** Primer sequences used in real-time PCR. **Figure S1.** Bioluminescent labeling of EVs. (**A-B**) Ex vivo imaging of Gluc-labeled EVs exhibited increasing bioluminescence signals with concentrations of EVs (R^2^ = 0.9907). **Figure S2.** DFLs isolation in vitro. (**A**) The image showed the separation of dermis (left) and epidermis (right) of newborn mice skin after Dispase II treatment. (**B**) Representative image showed fibroblasts crawled out from the edges of the skin dermal tissue by tissue-block cultivation. Scale bar, 200 μm. **Figure S3.** Quantitative statistical results of western blots. (**A**) Quantification of protein levels normalized to tubulin (*n* = 3; ^*^P < 0.05, ^**^P < 0.01 vs P2). **Figure S4.** CS-EVs treatment strategy in vivo. Schematic diagram for in vivo study. **Figure S5.** Features of aging skin and young skin tissue. (**A**) Representative skin sections of mouse at different ages, H&E and Masson trichrome stain. Scale bar, 50 μm. (**B**) The statistics of mean dermal or epidermal thickness as well as Collagen ratio at the indicated ages. Data are presented as the Mean ± SEM. (*n* = 5; ^**^P < 0.01 vs 12 W). **Figure S6.** Images of the uncropped immunoblots shown in the main figures. Boxes indicate cropped regions.

## Data Availability

All data generated and/or analyzed during this study are available from the corresponding author upon reasonable request.

## Abbreviations

ADSCs: Adipose-derived stem cells
β-GP: β-Glycerophosphate
CS: Chitosan
CS-EVs: CS hydrogel-incorporated EVs
DFLs: Dermal fibroblasts
DLS: Dynamic light scattering
ECM: Extracellular matrix
ELN: Elastin
EVs: Extracellular vesicles
FN: Fibronectin
GLuc: Gaussia luciferase
HE: Hematoxylin–eosin
MMPs: Matrix metalloproteinases
MSCs: Mesenchymal stem cells
MSC-EVs: MSC-derived extracellular vesicles
PBS: Phosphate-buffered saline
ROI: Region of interest
SA-β-gal: Senescence-associated β-galactosidase
SASP: Senescence-associated secretory phenotype
TEM: Transmission electron microscopy
TIMPs: Tissue inhibitor of metalloproteinases
