# Supplementary material for: Chitosan hydrogel-loaded MSC-derived extracellular vesicles promote skin rejuvenation by ameliorating the senescence of dermal fibroblasts
Source: Stem Cell Res Ther. 2021 Mar 20;12:196. doi: 10.1186/s13287-021-02262-4 (PMC7981922; doi:10.1186/s13287-021-02262-4)

**SUPPLEMENTAL DATA**

**Supplemental Table**

Table S1. Primer sequences used in real-time PCR

| Gene name | Primer Sequences |
| --- | --- |
| COL1 | Forward: GCCAAGAAGACATCCCTGAAG  Reverse: TGTGGCAGATACAGATCAAGC |
| COL3 | Forward: CCTGGCTCAAATGGCTCAC  Reverse: CAGGACTGCCGTTATTCCCG |
| Elastin | Forward: CTTTGGTTCCCCTGTCCCTG  Reverse: ATAAACCCAAAGAGCACACCAAC |
| Fibronectin | Forward: CAATGTCTACACCGTCCCTGA  Reverse: AGAAGGCTGCTGGAGTTGAAG |
| MMP-1 | Forward: AAGGTTAGCTTACTGTCACACGCTT  Reverse: CGACTCTAGAAACACAAGAGCAAGA |
| MMP-2 | Forward: CCAACTACGATGATGAC  Reverse: ACCAGTGTCAGTATCAG |
| MMP-3 | Forward: TTAAAGACAGGCACTTTTGGCG  Reverse: CCCTCGTATAGCCCAGAACT |
| MMP-9 | Forward: CCCGGACCAAGGATACAG  Reverse: GGCTTTCTCTCGGTACTG |
| TIMP-1 | Forward: GCAACTCGGACCTGGTCATAA  Reverse: CGGCCCGTGATGAGAAACT |
| TIMP-2 | Forward: TCAGAGCCAAAGCAGTGAGC  Reverse: GCCGTGTAGATAAACTCGATGTC |
| TIMP-3 | Forward: CTTCTGCAACTCCGACATCGT  Reverse: GGGGCATCTTACTGAAGCCTC |
|  |  |

**
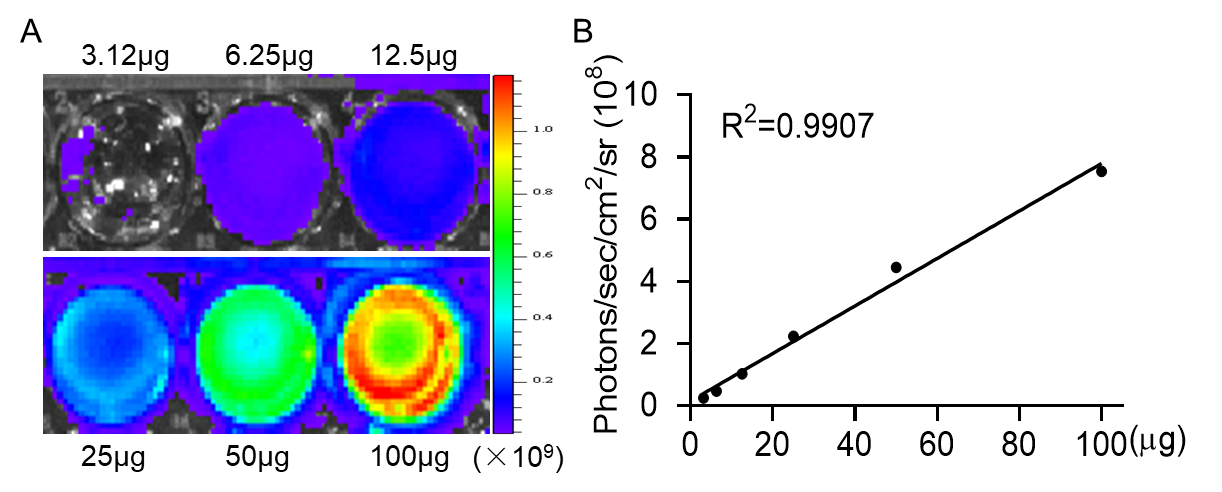
**S**upplemental Figures & Legend**

**Figure S1. Bioluminescent labeling of EVs.** (**A-B**) Ex vivo imaging of Gluc-labeled EVs exhibited increasing bioluminescence signals with concentrations of EVs (R^2^=0.9907).


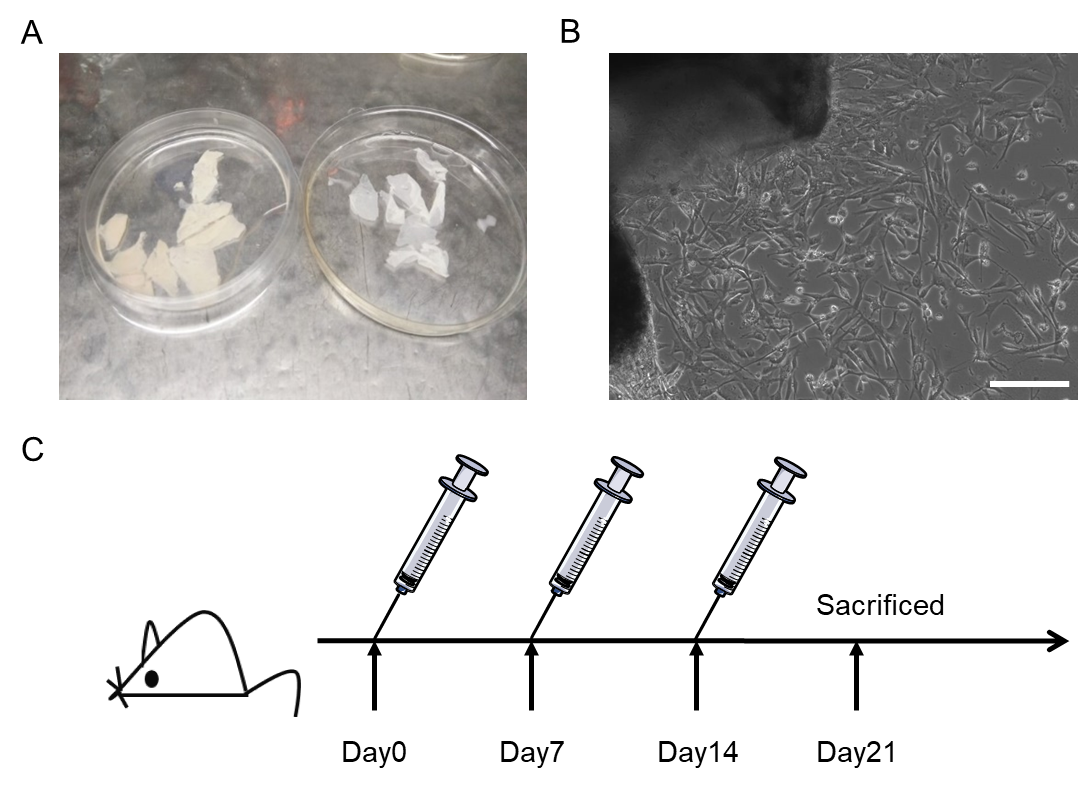
**Figure S2. DFLs isolation in vitro.** (**A**) The image showed the separation of dermis (left) and epidermis (right) of newborn mice skin after Dispase II treatment. (**B**) Representative image showed fibroblasts crawled out from the edges of the skin dermal tissue by tissue-block cultivation. Scale bar, 200 µm.

Figure S3. Quantitative statistical results of western blots. (**A**) Quantification of protein levels normalized to tubulin (n = 3; ^*^*P* <0.05, ^**^*P* <0.01 vs P2).
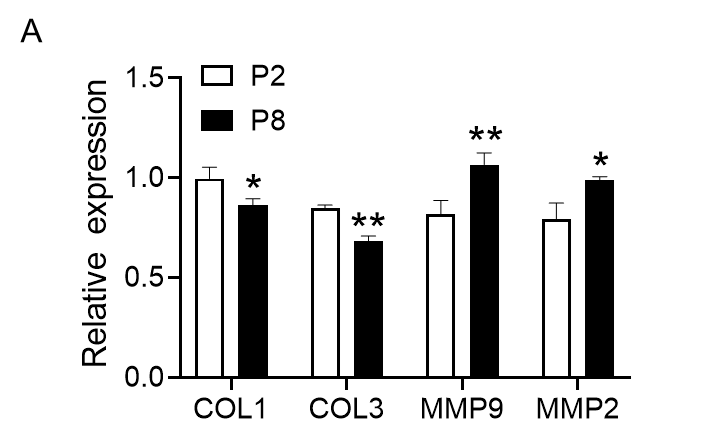


**
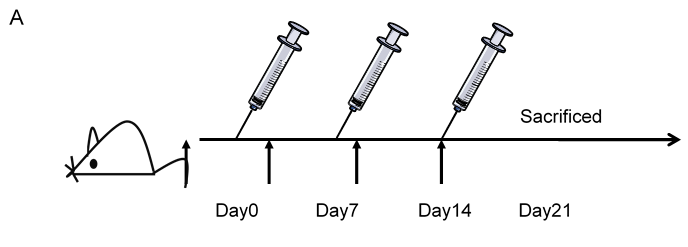
Figure S4: CS-EVs treatment strategy *in vivo*.** Schematic diagram for *in vivo* study.


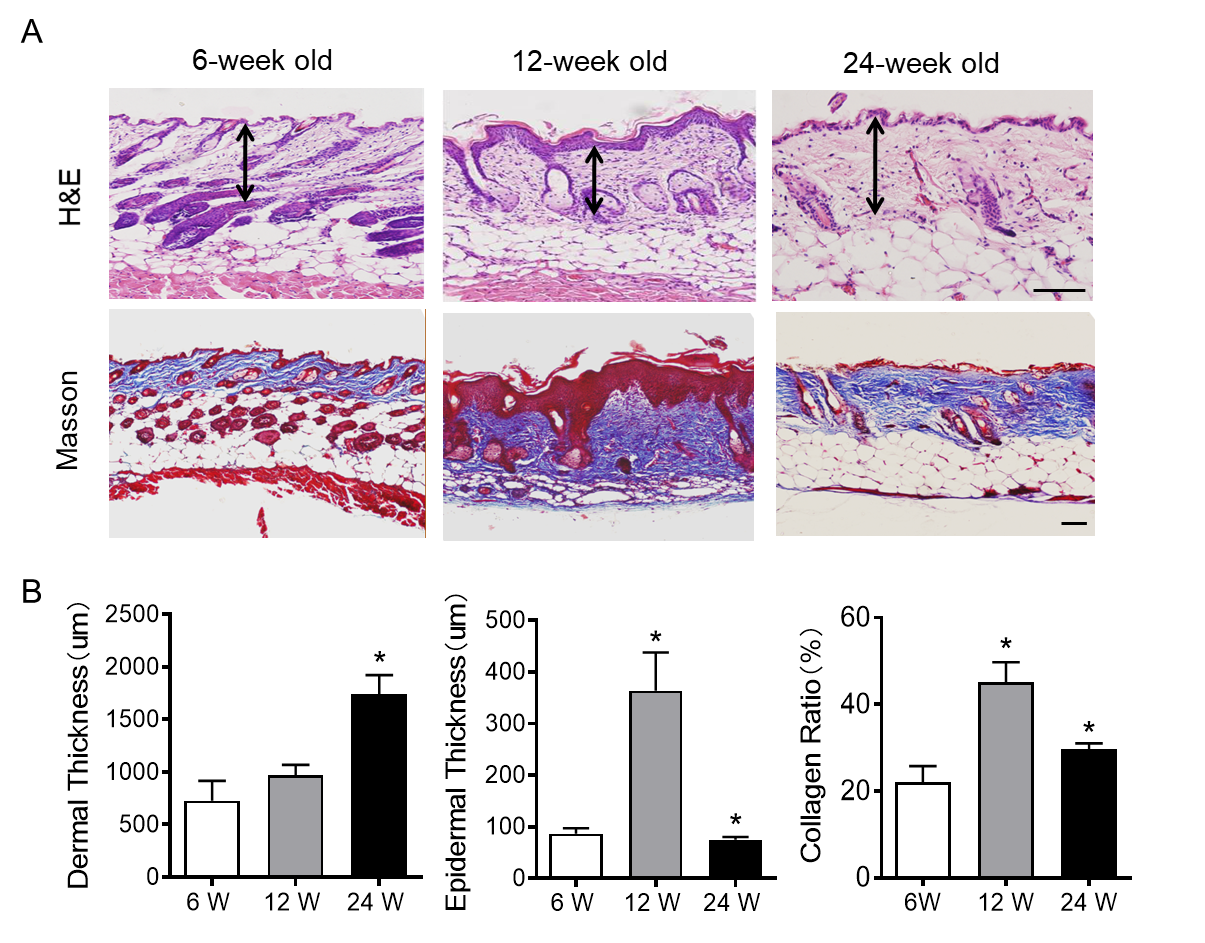
**Figure S5. Features of aging skin and young skin tissue.** (**A**) Representative skin sections of mouse at different ages, H&E and Masson trichrome stain. Scale bar, 50 µm. (**B**) The statistics of mean dermal or epidermal thickness as well as Collagen ratio at the indicated ages. Data are presented as the Mean ± SEM. (n = 5; ^**^*P*<0.01 vs 12W).

**Figure S6. Images of the uncropped immunoblots shown in the main figures.** Boxes indicate cropped regions.
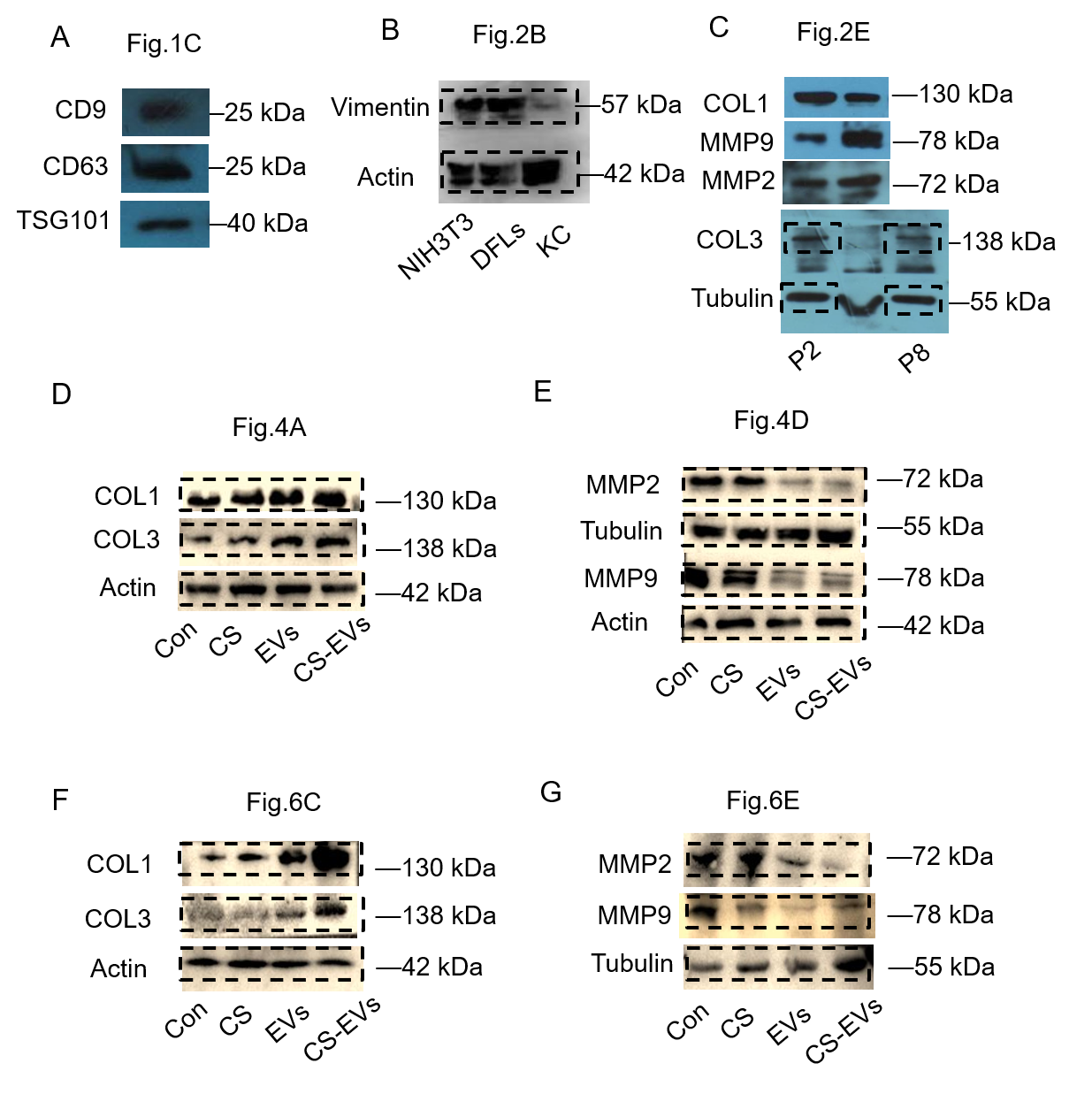

Supplement: Supplementary file 1 — Additional file 1: Table S1. Primer sequences used in real-time PCR. Figure S1. Bioluminescent labeling of EVs. (A-B) Ex vivo imaging of Gluc-labeled EVs exhibited increasing bioluminescence signals with concentrations of EVs (R2 = 0.9907). Figure S2. DFLs isolation in vitro. (A) The image showed the separation of dermis (left) and epidermis (right) of newborn mice skin after Dispase II treatment. (B) Representative image showed fibroblasts crawled out from the edges of the skin dermal tissue by tissue-block cultivation. Scale bar, 200 μm. Figure S3. Quantitative statistical results of western blots. (A) Quantification of protein levels normalized to tubulin (n = 3; *P < 0.05, **P < 0.01 vs P2). Figure S4. CS-EVs treatment strategy in vivo. Schematic diagram for in vivo study. Figure S5. Features of aging skin and young skin tissue. (A) Representative skin sections of mouse at different ages, H&E and Masson trichrome stain. Scale bar, 50 μm. (B) The statistics of mean dermal or epidermal thickness as well as Collagen ratio at the indicated ages. Data are presented as the Mean ± SEM. (n = 5; **P < 0.01 vs 12 W). Figure S6. Images of the uncropped immunoblots shown in the main figures. Boxes indicate cropped regions. [file 13287_2021_2262_MOESM1_ESM.docx]
